# Putting the pencil down

**DOI:** 10.1093/jvimsj/aalag257

**Published:** 2026-11-04

**Authors:** Steve DiBartola

**Affiliations:** Co-Editor-in-Chief, Journal of Veterinary Internal Medicine

*… perfection is finally attained not when there is no longer anything to add, but when there is no longer anything to take away …* - Antoine de saint-Exupéry

My first experience with professional writing occurred in 1977 when I was a small animal intern at Cornell University. I’d noticed it was common to see mildly to moderately increased creatine kinase activity on biochemical profiles of patients we saw in the clinic, so I decided to review their medical records and see if I could find some explanation for this observation. After collating my results, I wrote my paper and *mailed it* to a popular veterinary journal. I admired a visiting instructor from Australia and asked if he’d read my paper. Afterward, he said, “This is dreadful. You can’t submit it like this.” I sheepishly admitted I already had, and his response was, “Well, I’m sure the editors will rip it to shreds and give you a lot to think about when they reject it.” A few weeks later, I received a letter saying that the paper had been accepted. In my entire career, that has only happened one other time. Nearly always, submitted manuscripts go through several rounds of review and revision before achieving acceptance.

My interest in editing began in the early 1980s when I had to contend with the corrections Dr. Al Koltveit (editor at the American Veterinary Medical Association) made to my manuscripts. I couldn’t understand why he insisted upon certain revisions, and I sometimes *called him on the phone* to ask about them. I managed to put my ego aside and listened to what he had to say. I believe it made me a better writer. Today, after having edited 3000 manuscripts during my 30 years as co-editor-in-chief of the Journal, I’ve learned a lot about how my colleagues all over the world write—how they describe their work and its importance.

Scientific writing is fundamentally different from creative writing. Creative writing encompasses the entire range of human emotions and experience, whereas scientific writing presents verifiable information about specific scientific or clinical findings. As physicist Paul Dirac said, “In science one tries to tell people, in such a way as to be understood by everyone, something that no one ever knew before. But in poetry, it’s the exact opposite.”

Writing is hard work, and concern about final form can contribute to writer’s block. A reasonable strategy is to just sit down and capture your initial thoughts in a rough draft. Thrive on revision, because repeated editing is fundamental to good writing. Edit yourself relentlessly. You’re probably done when you find yourself changing something in draft 10 back to the way it was in draft 9. Follow a logical progression of thought and express yourself clearly and concisely. Conciseness is important even in today’s world of electronic publication, where word counts theoretically no longer matter. Your audience consists of busy professionals who have limited time to read.

Ask a colleague who knows your work and has good writing skills to read your paper and provide feedback. Welcome constructive criticism—a paper covered in corrections, comments, and suggestions will help you more than a smiley face at the top of the draft followed by “looks good.”

Some authors may have been frustrated by my heavy-handed editing, but I hope at least some have matured as writers based on the editorial changes I’ve made to their papers. I do have some regrets. Being meticulous is one thing, but being compulsive and inflexible is rarely helpful. My approach was informed by my belief that a journal should have its own recognizable style.

You may argue that the quality of your writing is not important as long as the reader knows what you are trying to say, but if your writing is careless a reader may conclude that the work it describes was similarly flawed. Making an effort to express oneself well is important. Conscientious editorial oversight ensures publication of high-quality manuscripts and a consistent style for a journal.

Scientific publishing is facing several challenges today. I believe diligent review of submitted manuscripts by subject matter experts remains crucial to publication of high-quality papers. Finding qualified reviewers willing to conduct critical reviews punctually is becoming increasingly difficult because of exploding numbers of manuscript submissions. For each manuscript, editors must send numerous invitations before securing the necessary number of reviewers, and many reviews are returned long after the deadline. Programs to train and mentor potential reviewers early in their careers could be helpful, but such programs require resources to organize and administer. Likewise, formal recognition and academic credit for peer review in university promotion and tenure processes would be helpful.

Although artificial intelligence (AI) could be viewed as a potential solution for the shortage of human scientific reviewers, most journals prohibit the use of AI for reviewing manuscripts. Uploading an unpublished manuscript to a chatbot violates editorial confidentiality and compromises the author’s intellectual property. Furthermore, any inaccurate content uploaded and used for training of large language models could result in flawed information being recycled into future AI output, potentially compromising the scientific literature. Reviews produced by AI superficially may appear well-balanced, but typically lack critical assessment of methodology and research findings. Editors can inform reviewers that AI is not to be used, but enforcing this prohibition is challenging.

The tendency of AI to ingratiate itself with users, hallucinate non-existent references, and generate superficial, uncritical analysis are reasons why it should not be used in the initial writing of a paper. It can, however, assist authors in editing drafts of their papers for grammar and clarity, and assure that a manuscript meets the target journal’s editorial style guidelines. A human author should review and make any necessary edits to the final draft before it is submitted. AI cannot be listed as an author of a paper because authorship requires accountability for content. All authors on a paper must be able to defend the paper publicly, and AI cannot meet this requirement. Authors must use AI responsibly and disclose how it was used in writing the paper. As is the case for disclosure of conflicts of interest, editors can require authors to indicate at the time of submission whether or not AI was used in writing the manuscript, but cannot assess the veracity of their responses.

My co-editor-in-chief Ken Hinchcliff and I have had a long rewarding partnership. I believe our different personalities and approaches to the challenges of maintaining a high-quality veterinary journal complemented each other well. Ken is innovative and always thinking of ways to keep the Journal at the forefront of veterinary publishing. In 2014, he wanted to move the Journal to full open access and publication in electronic format only. I was hesitant but deferred to Ken’s vision, and he was absolutely right.

I also extend my appreciation to Ivy Leventhal for many years of steadfast support of the Journal’s authors and editors and to Jenny Brown for assuming increasing responsibility for central administration of the Journal. Also, I’ve had the opportunity to collaborate with many talented associate editors over the past 30 years. It’s been a pleasure to sit down with them at editorial board meetings to discuss the Journal and get to know one another personally. I definitely will miss the entire experience of serving as co-editor-in-chief.

The Journal was inaugural editor Al Legendre’s brainchild, and when I assumed my role Al said, “Don’t hurt my baby.” I hope I have nurtured the baby and helped it mature into the reliable and respected journal it is today. I am confident the Journal will continue to grow and thrive for many years to come.

**Figure:**
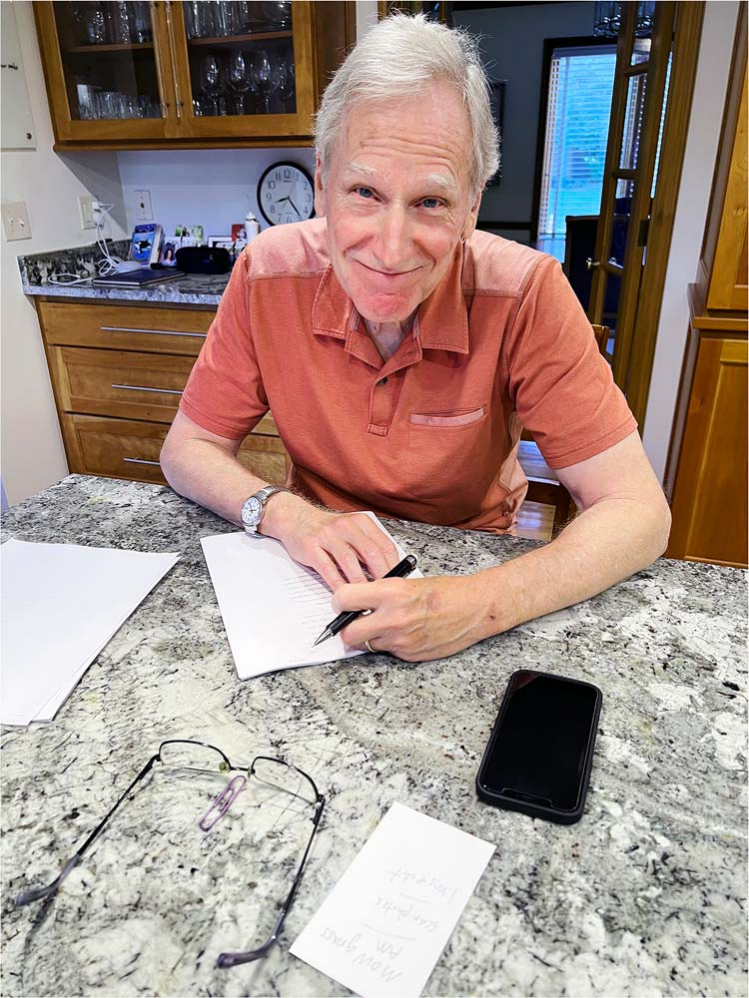
Steve DiBartola editing a manuscript.

